# Racial and Ethnic Disparity in Approach for Pediatric Intensive Care Unit Research Participation

**DOI:** 10.1001/jamanetworkopen.2024.11375

**Published:** 2024-05-15

**Authors:** Sarah L. Mayer, Michelle R. Brajcich, Lionola Juste, Jesse Y. Hsu, Nadir Yehya

**Affiliations:** 1 Department of Anesthesiology and Critical Care Medicine, Children’s Hospital of Philadelphia and University of Pennsylvania, Philadelphia; 2 Center for Clinical Epidemiology and Biostatistics, Perelman School of Medicine, University of Pennsylvania, Philadelphia; 3 Leonard Davis Institute of Health Economics, University of Pennsylvania, Philadelphia

## Abstract

**Importance:**

While disparities in consent rates for research have been reported in multiple adult and pediatric settings, limited data informing enrollment in pediatric intensive care unit (PICU) research are available. Acute care settings such as the PICU present unique challenges for study enrollment, given the highly stressful and emotional environment for caregivers and the time-sensitive nature of the studies.

**Objective:**

To determine whether race and ethnicity, language, religion, and Social Deprivation Index (SDI) were associated with disparate approach and consent rates in PICU research.

**Design, Setting, and Participants:**

This retrospective cohort study was performed at the Children’s Hospital of Philadelphia PICU between July 1, 2011, and December 31, 2021. Participants included patients eligible for studies requiring prospective consent. Data were analyzed from February 2 to July 26, 2022.

**Exposure:**

Exposures included race and ethnicity (Black, Hispanic, White, and other), language (Arabic, English, Spanish, and other), religion (Christian, Jewish, Muslim, none, and other), and SDI (composite of multiple socioeconomic indicators).

**Main Outcomes and Measures:**

Multivariable regressions separately tested associations between the 4 exposures (race and ethnicity, language, religion, and SDI) and 3 outcomes (rates of approach among eligible patients, consent among eligible patients, and consent among those approached). The degree to which reduced rates of approach mediated the association between lower consent in Black children was also assessed.

**Results:**

Of 3154 children included in the study (median age, 6 [IQR, 1.9-12.5] years; 1691 [53.6%] male), rates of approach and consent were lower for Black and Hispanic families and those of other races, speakers of Arabic and other languages, Muslim families, and those with worse SDI. Among children approached for research, lower consent odds persisted for those of Black race (unadjusted odds ratio [OR], 0.73 [95% CI, 0.55-0.97]; adjusted OR, 0.68 [95% CI, 0.49-0.93]) relative to White race. Mediation analysis revealed that 51.0% (95% CI, 11.8%-90.2%) of the reduced odds of consent for Black individuals was mediated by lower probability of approach.

**Conclusions and Relevance:**

In this cohort study of consent rates for PICU research, multiple sociodemographic factors were associated with lower rates of consent, partly attributable to disparate rates of approach. These findings suggest opportunities for reducing disparities in PICU research participation.

## Key Points

**Question:**

Are sociodemographic factors associated with rates of approach and consent for pediatric intensive care unit (PICU) research?

**Findings:**

This cohort study of 3154 children found disparities in approach and consent according to race and ethnicity, language, religion, and degree of social deprivation. Lower consent rates were partly mediated by lower approach rates, with reduced approach mediating approximately half of the lower rates of consent for Black children.

**Meaning:**

In this study, multiple sociodemographic variables were associated with disparate consent rates for PICU research, and strategies to increase approaches could contribute to equitable enrollment in PICU studies.

## Teaser

This cohort study assesses all interventional and observational research studies that required prospective informed consent from a large academic pediatric intensive care unit (PICU) over 10 years and analyzes disparities in approach and consent by race and ethnicity, religion, spoken language, and socioeconomic status.

## Introduction

Inclusive representation in research is important for ensuring generalizability of results, equitable access to medical advances, and improved trust between patients and clinicians. Disparities in research enrollment have been demonstrated in oncology,^1,2,3^ COVID-19 trials,^4,5^ and the general adult population.^6,7^ Importantly, racial and ethnic disparities in research are often indexed to census data,^8,9,10,11,12^ which may differ from the population eligible for studies. In US pediatric trials from 2011 to 2020, relative to census demographics, Black children appear overrepresented, whereas American Indian and Alaska Native, Asian, and Native Hawaiian and Other Pacific Islander children appear to be underrepresented,^11^ and no racial or ethnic disparities were identified in pediatric drug or device studies.^9^ However, defining overrepresentation relative to census data masks actual disparities in consent rates among eligible participants, given higher rates of hospital^13^ and pediatric intensive care unit (PICU)^14,15,16^ admission for Black children and residents of high-poverty neighborhoods. In addition to race and ethnicity, disparities in enrollment have also been demonstrated based on language preference,^17,18,19^ although this has been less studied in critical care.

The PICU presents unique challenges for study enrollment, given the highly stressful and emotional environment for caregivers and the time-sensitive nature of enrolling participants with severe and rapidly changing disease.^20,21^ This limits a research team’s ability to build trust and rapport with a family prior to study introduction and restricts families’ time to consider a study prior to consenting. Research investigating disparities in study enrollment in the PICU is limited to a reanalysis of a cluster-randomized interventional trial of sedation management (Randomized Evaluation of Sedation Titration for Respiratory Failure [RESTORE])^22^ and an evaluation of enrollment in a biorepository at a single center.^23^ Both studies identified lower rates of research approach and consent of patients who were members of racial and ethnic minority groups. These results contrast with the conclusions of studies referencing census data, and further work is necessary to determine whether these disparities are seen across a larger sample of pediatric critical care research.

Therefore, we analyzed all research studies, interventional and observational, from a large academic PICU over 10 years that required prospective informed consent. We hypothesized that disparities in study approach and consent existed according to race and ethnicity, religion, spoken language, and socioeconomic status and that disparities in consent rate were partly mediated by the probability of approaching families to offer study participation.

## Methods

### Design and Setting

This retrospective cohort study reviewed all screening and consent logs for all research studies prospectively enrolling in the Children’s Hospital of Philadelphia (CHOP) PICU from July 1, 2011, to December 31, 2021. The CHOP Institutional Review Board reviewed this study and provided an exempt determination from approval and informed consent because it was a retrospective cohort. The study was reported in accordance with the Strengthening the Reporting of Observational Studies in Epidemiology (STROBE) guideline. Additional details are provided in the eMethods in Supplement 1.

### Eligibility

All patients eligible for research requiring consent were potentially eligible for this study. Protocols for each study were examined for specific inclusion and exclusion criteria to determine eligibility for our study. Detailed eligibility criteria are provided in the eMethods in Supplement 1.

### Definitions

Screening logs were linked to the electronic medical record (EMR) for data collection. We examined 4 distinct exposures: race and ethnicity, preferred language, religion, and Social Deprivation Index (SDI). Since our EMR permits Hispanic to be reported as either a race or ethnicity, we combined race and ethnicity into groupings of Hispanic, non-Hispanic Black, non-Hispanic White, and other (including American Indian or Alaska Native, Asian, Native Hawaiian or Other Pacific Islander, Indian, multiracial, other, and refused). Preferred language was encoded as Arabic, English, Spanish, and other. Religion was coded as Christian, Jewish, Muslim, none, and other. Zip code was used to assign the SDI, a validated composite of area-level deprivation extracted from the American Community Survey.^24^

We modeled 3 distinct outcomes: approach for research, consent to research among eligible patients, and consent to research among those approached. Confounders included age, sex, PICU length of stay prior to screening, illness severity as defined by Pediatric Risk of Mortality (PRISM) III score at 12 hours (range, 0-74, with higher scores indicating greater mortality risk), year of screening, elective or nonelective admission, origin of admission (emergency department, inpatient floor, neonatal ICU, operating room, or outside hospital), and study type (observational or interventional). Entries reflecting the same patient eligible for different trials or on separate admissions were retained as separate encounters. Quantitative variables were treated as continuous variables.

### Statistical Analysis

Data were analyzed from February 2 to July 26, 2022. Separate logistic regression models were used to separately test the association between the 4 exposures of interest (race and ethnicity, language, religion, and SDI) and the 3 outcomes (approach for study [all eligible patients], consent to study [all eligible patients], and consent if approached [restricted to those approached]). All analyses (unadjusted and adjusted) used robust variance estimators to account for 2-way nonnested clustering (by patient and by study), and all multivariable analyses were adjusted for confounders selected using a causal framework. Exposures had less than 5% missingness except for language (187 [5.9%]), and as the nonrandom missingness of language made imputation conceptually difficult with the available variables,^25^ only complete case analyses were conducted in the primary analyses. Exposures were analyzed in independent models, given the complex interactions and potential collinearity among race and ethnicity, language, religion, and SDI (eFigure 1 in Supplement 1). We performed multiple additional analyses. First, given possible differences between observational and interventional studies, we a priori tested for differential associations between exposures and outcomes according to study type. Second, to test whether data missingness affected our conclusions, we repeated analyses using multiple imputation by chained equations^26^ (10 imputations over the entire cohort) to impute missing values for language, religion, and SDI. Third, we performed an exploratory analysis by including all exposure variables in the model, in addition to confounders, on the dataset with imputed missing data. Fourth, as an alternative method to model the data, we performed multinomial regression for odds of approach and declining consent and approach and providing consent, with not approached set as the reference.

Finally, causal mediation analysis^27^ was performed to estimate the degree to which the association between Black race and odds of consent was mediated by the probability of being approached. This was a 2-step procedure where we first estimated the probability of being approached for all participants using a separate logistic regression model with all variables included as independent variables and being approached as the outcome. We then used this estimated probability of being approached as a mediator of the association between Black (compared with White) race and odds of consent. All analyses were conducted in Stata, version 18 (StataCorp LLC), with 2-sided *P* < .05 considered significant for main analyses and 2-sided *P* < .10 for assessing the significance of interaction terms.

## Results

### Description of the Cohort

Forty-four screening logs from studies (10 interventional trials and 34 observational studies) enrolling between 2011 and 2021 included 35 837 encounters. Two studies had no patients screened. Of the total, 31 585 encounters were excluded as ineligible according to the eligibility criteria for the parent studies, 180 for incomplete or ambiguous data regarding eligibility, 299 for suspended enrollment (including during the COVID-19 pandemic), 561 for patient location in the cardiac ICU (rather than PICU), and 58 for inability to determine whether or not a patient was approached (ie, unable to assign a primary outcome), leaving a total of 3154 patients eligible for our study (eFigure 2 in Supplement 1). Of these, sex was recorded as male for 1691 patients (53.6%) and female for 1461 (46.4%), with data missing for 2 (0.1%). Median age was 6.0 (IQR, 1.9-12.5) years, and median PRISM III score was 9 (IQR, 4-15). In terms of race and ethnicity, 855 patients (27.1%) were identified in the EMR as Black, 484 (15.3%) as Hispanic, 1204 (38.2%) as White, and 611 (19.4%) as other. English was the preferred language for most patients (2635 of 2967 with data available [88.8%]). Of the 3154 eligible patients, 896 patients were not approached, 816 were approached but declined consent, and 1442 (45.7% of eligible patients and 63.9% of approached patients) consented to studies (Table).

**Table.** Description of the Cohort

Variable	Patient group^a^			
	All (N = 3154)	Not approached (n = 896)	Approached	
			Declined (n = 816)	Consented (n = 1442)
Interventional trial	844 (26.8)	277 (30.9)	246 (30.1)	321 (22.3)
Prescreen PICU days, median (IQR)	1 (0-1)	1 (0-2)	1 (0-1)	1 (1-1)
PRISM III score, median (IQR)^b^	9 (4-15)	9 (3-15)	9 (4-15)	9 (4-15)
Age, median (IQR), y	6.0 (1.9-12.5)	4.8 (1.5-12)	7.0 (1.9-13)	6.5 (2.2-12.4)
Recorded sex				
Female	1461 (46.4)	406 (45.3)	396 (48.5)	659 (45.7)
Male	1691 (53.6)	489 (54.6)	420 (51.5)	782 (54.2)
Unknown	2 (0.1)	1 (0.1)	0	1 (0.1)
Elective admission	283 (9.0)	86 (9.6)	70 (8.6)	127 (8.8)
Patient origin				
ED	1198 (38.0)	335 (37.4)	295 (36.2)	568 (39.4)
Floor	577 (18.3)	148 (16.5)	179 (21.9)	250 (17.3)
Neonatal ICU	57 (1.8)	26 (29.0)	11 (1.3)	20 (1.4)
Operating room	361 (11.4)	100 (11.2)	95 (11.6)	166 (11.5)
Outside hospital	961 (30.5)	287 (32.0)	236 (28.9)	438 (30.4)
Race and ethnicity				
Black (non-Hispanic)	855 (27.1)	258 (28.8)	253 (31.0)	344 (23.9)
Hispanic	484 (15.3)	153 (17.1)	102 (12.5)	229 (15.9)
White (non-Hispanic)	1204 (38.2)	254 (28.3)	321 (39.3)	629 (43.6)
Other^c^	611 (19.4)	231 (25.8)	140 (17.2)	240 (16.6)
Language^d^				
English	2635 (88.8)	666 (79.8)	706 (92.9)	1263 (92.1)
Spanish	151 (5.1)	56 (6.7)	26 (3.4)	69 (5.0)
Arabic	107 (3.6)	59 (7.1)	17 (2.2)	31 (2.3)
Other	74 (2.5)	54 (6.5)	11 (1.4)	9 (0.7)
Religion^e^				
None	726 (23.6)	187 (21.7)	192 (24.0)	347 (24.6)
Christian	1634 (53.2)	429 (49.8)	420 (52.6)	785 (55.6)
Muslim	293 (9.5)	132 (15.3)	62 (7.8)	99 (7.0)
Jewish	117 (3.8)	21 (2.4)	48 (6.0)	48 (3.4)
Other	301 (9.8)	92 (10.7)	77 (9.6)	132 (9.4)
SDI (IQR)^f^	43 (18-83)	53 (24-85)	41 (18-82)	40 (17-82)

Abbreviations: ED, emergency department; ICU, intensive care unit; PICU, pediatric ICU; PRISM, Pediatric Risk of Mortality; SDI, Social Deprivation Index.

^a^ Unless otherwise indicated, data are expressed as No. (%) of patients.

^b^ Scores range from 0 to 74, with higher scores indicating greater mortality risk.

^c^ Includes American Indian or Alaska Native, Asian, Native Hawaiian or Other Pacific Islander, Indian, multiracial, other, and refused.

^d^ Data were available for 2967 patients.

^e^ Data were available for 3071 patients.

^f^ Ranges from 0 (least deprived) to 100 (most deprived) and was divided by 10 for reporting. Data were available for 3024 patients.

### Probability of Approach

**Figure 1.**
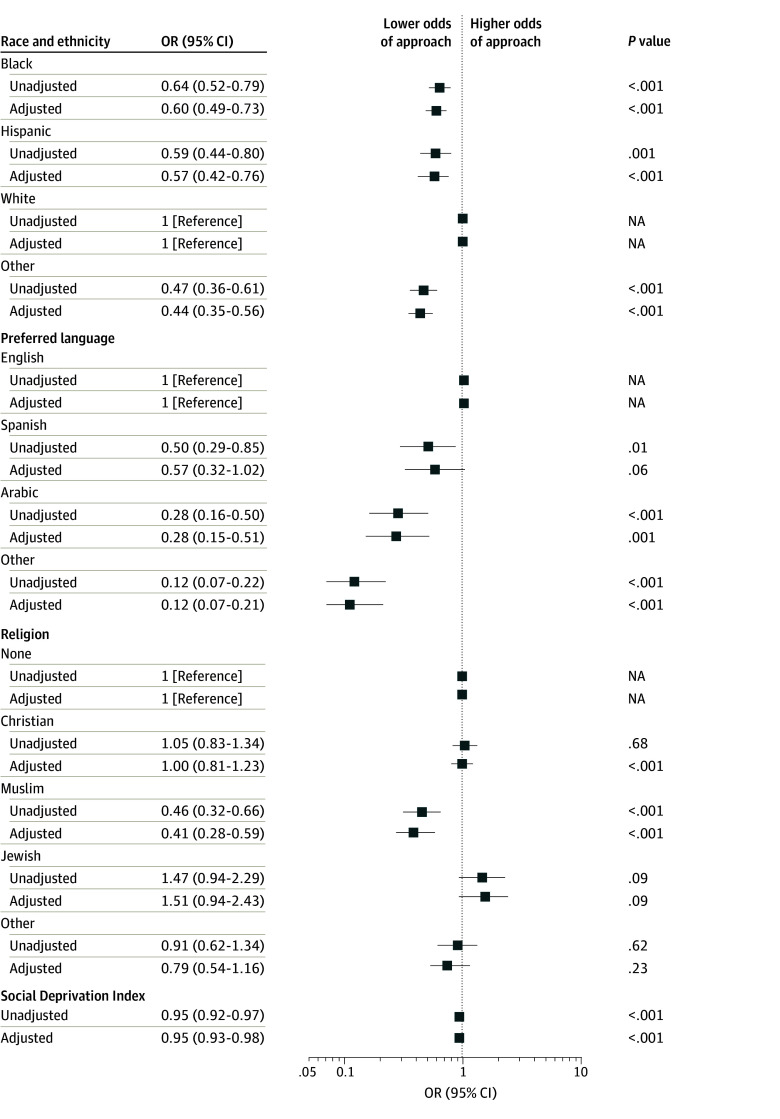
Unadjusted and Adjusted Odds of Approach for Study Participation According to Race and Ethnicity, Preferred Language, Religion, or Social Deprivation Index Social Deprivation Index ranges from 0 (least deprived) to 100 (most deprived) and was divided by 10 for reporting. Adjustments were for age, sex, pediatric intensive care unit length of stay before screening date, Pediatric Risk of Mortality III score, year of screening, elective or nonelective admission, origin of admission, and study type (observational or interventional). Other race included American Indian or Alaska Native, Asian, Native Hawaiian or Other Pacific Islander, multiracial, other, and refused. NA indicates not applicable; OR, odds ratio.

Relative to White children, lower odds of approach were seen for Black children (unadjusted odds ratio [OR], 0.64 [95% CI, 0.52-0.79]; adjusted OR [AOR], 0.60 [95% CI, 0.49-0.73]), Hispanic children (OR, 0.59 [95% CI, 0.44-0.80]; AOR, 0.57 [95% CI, 0.42-0.76]), and children of other race (OR, 0.47 [95% CI, 0.36-0.61]; AOR, 0.44 [95% CI, 0.35-0.56]) (Figure 1 and eTable 1 in Supplement 1). Black and Hispanic families were more commonly not approached due to family unavailability, while Hispanic families and families of other race were more commonly not approached due to perceived language barriers (eTable 2 in Supplement 1).

Compared with families who preferred English (eTable 1 in Supplement 1), families who preferred Arabic (OR, 0.28 [95% CI, 0.16-0.50]; AOR, 0.28 [95% CI, 0.15-0.51]), Spanish (OR, 0.50 [95% CI, 0.29-0.85]; AOR, 0.57 [95% CI, 0.32-1.02]), or other language (OR, 0.12 [95% CI, 0.07-0.22]; AOR, 0.12 [95% CI, 0.07-0.21]) had lower odds for approach, primarily due to perceived language barriers (eTable 3 in Supplement 1). Muslim families had lower odds for approach than those with none for religious affiliation (OR, 0.46 [95% CI, 0.32-0.66]; AOR, 0.41 [95% CI, 0.28-0.59]), also primarily due to language barriers (eTable 4 in Supplement 1). Higher (worse) SDI was associated with lower odds of approach (OR, 0.95 [95% CI, 0.92-0.97] per 10-point change; AOR, 0.95 [95% CI, 0.93-0.98] per 10-point change).

In stratified analysis, odds of approach were more favorable for other language for interventional (OR, 0.25 [95% CI, 0.08-0.84]) rather than observational (OR, 0.09 [95% CI, 0.05-0.16]) studies (*P* = .09 for interaction) (eTable 5 in Supplement 1). No other variables had a differential association with approach according to study type.

### Probability of Consent Among All Eligible Patients

**Figure 2.**
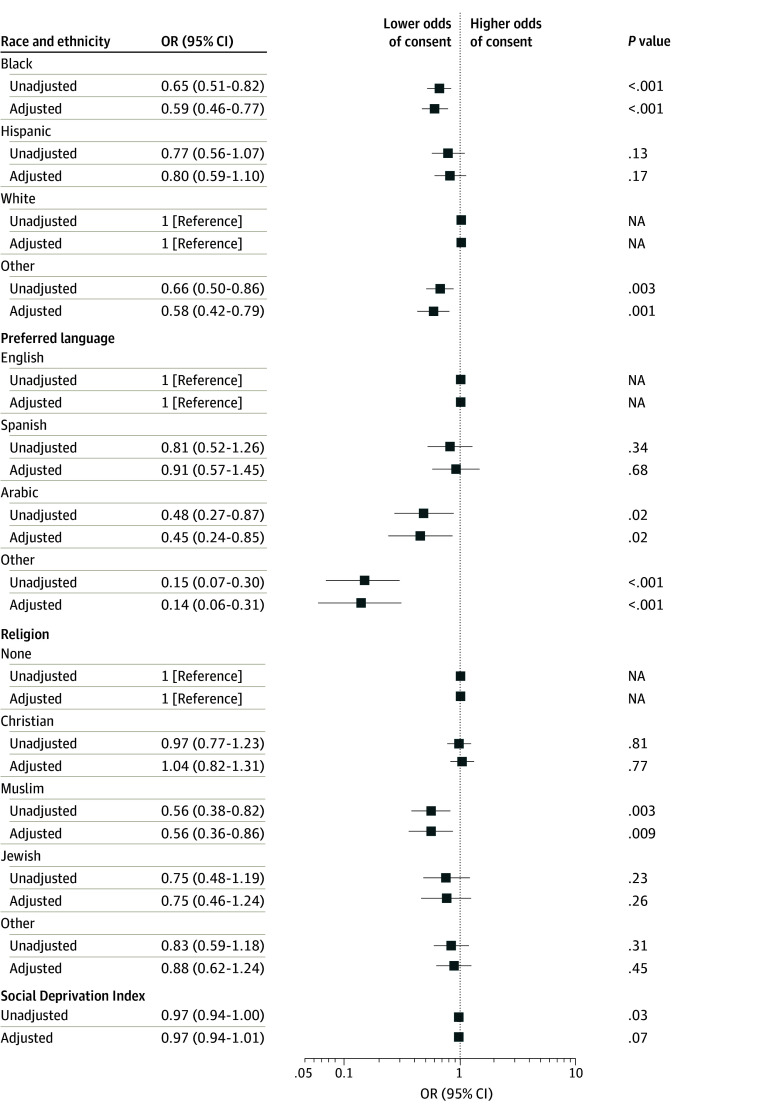
Unadjusted and Adjusted Odds of Consent Among All Eligible Patients According to Race and Ethnicity, Preferred Language, Religion, or Social Deprivation Index Social Deprivation Index ranges from 0 (least deprived) to 100 (most deprived) and was divided by 10 for reporting. Adjustments were for age, sex, pediatric intensive care unit length of stay before screening date, Pediatric Risk of Mortality III score, year of screening, elective or nonelective admission, origin of admission, and study type (observational or interventional). Other race included American Indian or Alaska Native, Asian, Native Hawaiian or Other Pacific Islander, multiracial, other, and refused. NA indicates not applicable; OR, odds ratio.

Among eligible patients, Black children (OR, 0.65 [95% CI, 0.51-0.82]; AOR, 0.59 [95% CI, 0.46-0.77]) and those of other race (OR, 0.66 [95% CI, 0.50-0.86]; AOR, 0.58 [95% CI, 0.42-0.79]) had lower consent odds (Figure 2 and eTable 6 in Supplement 1) relative to White children. Families preferring Arabic (OR, 0.48 [95% CI, 0.27-0.87]; AOR, 0.45 [95% CI, 0.24-0.85]) or other language (OR, 0.15 [95% CI, 0.07-0.30]; AOR, 0.14 [95% CI, 0.06-0.31]) were less likely to consent relative to English-speaking families. Muslim families also were less likely to consent (OR, 0.56 [95% CI, 0.38-0.82]; AOR, 0.56 [95% CI, 0.36-0.86]) relative to those with none for religious affiliation. Higher (worse) SDI had an OR less than 1 for odds of consent, but the results were not significant in adjusted analysis (OR, 0.97 [95% CI, 0.94-1.00] per 10-point change; AOR, 0.97 [95% CI, 0.94-1.01] per 10-point change). In stratified analysis, odds of consent were more favorable for interventional vs observational studies for other race (ORs, 0.83 [95% CI, 0.62-1.12] and 0.51 [95% CI, 0.35-0.74], respectively; *P* = .009 for interaction) and other language (ORs, 0.44 [95% CI, 0.14-1.41] and 0.09 [95% CI, 0.04-0.21], respectively; *P* = .03 for interaction) (eTable 7 in Supplement 1).

### Probability of Consent Among Approached Patients

**Figure 3.**
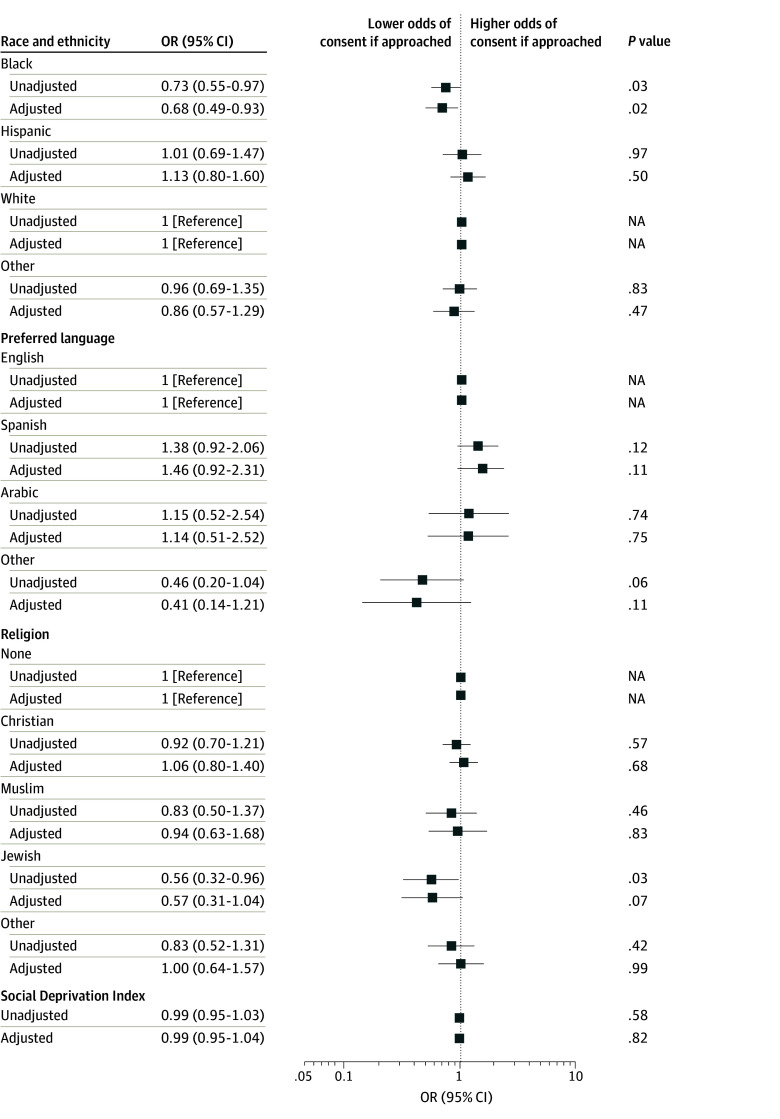
Unadjusted and Adjusted Odds of Consent Among Approached Patients According to Race and Ethnicity, Preferred Language, Religion, or Social Deprivation Index Social Deprivation Index ranges from 0 (least deprived) to 100 (most deprived) and was divided by 10 for reporting. Adjustments were for age, sex, pediatric intensive care unit length of stay before screening date, Pediatric Risk of Mortality III score, year of screening, elective or nonelective admission, origin of admission, and study type (observational or interventional). Other race included American Indian or Alaska Native, Asian, Native Hawaiian or Other Pacific Islander, multiracial, other, and refused. NA indicates not applicable.

When restricted to patients approached for research participation, odds of consent did not differ by race, language, religion, or SDI, except for Black relative to White children (OR, 0.73 [95% CI, 0.55-0.97]; AOR, 0.68 [95% CI, 0.49-0.93]) and Jewish children relative to those with none for religious affiliation (OR, 0.56 [95% CI, 0.32-0.96]; AOR, 0.57 [95% CI, 0.31-1.04]) (Figure 3 and eTable 8 in Supplement 1). In stratified analysis, odds of consent were more favorable for interventional rather than observational studies for other race (ORs, 1.19 [95% CI, 0.72-1.96] and 0.76 [95% CI, 0.48-1.20], respectively; *P* = .07 for interaction) and other language (ORs, 1.24 [95% CI, 0.47-3.26] and 0.24 [95% CI, 0.07-0.87], respectively; *P* = .08 for interaction) (eTable 9 in Supplement 1).

### Supplementary Analyses

When we repeated the analyses after imputing missing language, religion, and SDI (eTable 10 in Supplement 1), we found similar conclusions as those of the primary analyses (compare Figure 1 with Figure 3). Overall effect sizes with imputed data were similar for associations with approach among all eligible patients, consent among all eligible patients, and consent among those approached (compared with eTables 1, 6, and 8 in Supplement 1).

In an exploratory analysis, we examined whether conclusions were substantially affected by our choice to model race and ethnicity, language, religion, and SDI separately. Using the fully imputed dataset, we explored all exposure variables in a single model (eTable 11 in Supplement 1). In this analysis, effect sizes for associations between demographic variables and odds of approach or consent among all patients were all somewhat attenuated toward the null, although overall conclusions did not change, with the same variables retaining statistical significance. When assessing the odds of consent among those approached, conclusions were unchanged, with Black race and Jewish religion associated with lower odds of consent among those approached, identical to our primary analyses (compared with Figure 3; eTables 8 and 10 in Supplement 1). Interestingly in this fully adjusted model, the ORs for Black race and Jewish religion were more extreme than in the primary analyses, although we caution that based on the assumptions laid out in our directed acyclic graph (eFigure 1 in Supplement 1), this analysis may have a biased interpretation.

Last, we explored multinomial regression as an alternative analytic method (eTable 12 in Supplement 1). All racial and ethnic minority patient groups (relative to White patients), all non–English-speaking patients (relative to English-speaking patients), and Muslim patients (relative to those with none for religious affiliation) had lower odds of approach overall and of consent relative to not being approached. Consistent with the primary analysis, Black race and Jewish religion had significant differences between the effect sizes reported for approached and declined and approached and consented.

### Mediation Analysis

**Figure 4.**
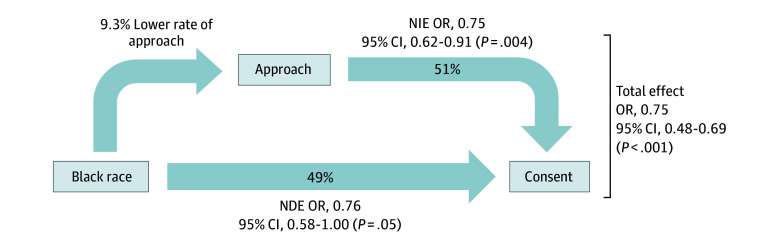
Mediation Analysis Framework Showing the Proposed Associations Between Black Race, Approach for Study Participation, and Probability of Consent It was hypothesized that reduced probability of approach would act as a mediator of the association between Black race and reduced consent. In this framework, we assessed the natural indirect effect (NIE) via mediation and the natural direct effect (NDE) of Black race (reference group, White race) on consent rates. Odds ratios (ORs) are presented, adjusted for age, sex, pediatric intensive care unit length of stay before screening date, Pediatric Risk of Mortality III score, year of screening, elective or nonelective admission, origin of admission, and study type (observational or interventional).

Given persistently lower odds of approach and consent for Black children in all of our analyses, causal mediation analysis was performed to determine the degree to which a lower estimated probability of being approached mediated overall lower rates of consent (Figure 4). We found that 51.0% (95% CI, 11.8%-90.2%) of the lower rates of consent for Black children were mediated by the lower rates of approach.

## Discussion

In children eligible for research studies from 2011 to 2021 in the CHOP PICU, this cohort study found underrepresentation according to race and ethnicity (Black, Hispanic, and other), preferred language (Arabic or other), religion (Jewish, Muslim, or other), and socioeconomic status (higher SDI). These disparities were primarily attributable to lower odds of being approached by research teams, with attenuation of ORs when analyzing only patients who were approached. Overall, our results suggest that improved rates of representative enrollment can be achieved with increased rates of research approach, including among Black children. However, there may be additional reasons why Black patients are less likely to consent even if approached that require additional investigation.

Our results are concordant with the existing 2 studies regarding consent disparities in the PICU, which showed lower consent in racial and ethnic minority groups and non-English speakers.^22,23^ Our study adds to this literature by attempting to quantify the degree to which research populations represent the population eligible for the study, rather than the US population as a whole. Our findings of lower odds of consent for Black children contrasts with some recent literature. In a review of 612 pediatric trials conducted between 2011 and 2020, Black patients were reported to be overrepresented relative to the US census (OR, 1.88 [95% CI, 1.87-1.89]),^11^ although this was not confirmed in a review of pediatric studies listed on ClinicalTrials.gov between 2007 and 2020.^12^ However, using the US census as a reference to identify disparate rates of trial enrollment can be problematic,^8,10,11^ as previous studies have demonstrated disparities according to race and ethnicity and SDI in patients admitted to PICUs.^15,16^ The magnitude of disparities in study enrollment can be further biased due to systematic undercounting of Black individuals in the US census.^28^ Even attempts to identify disparities in enrollment by referencing disease or hospitalization prevalence can be inaccurate,^12^ as not every patient with a diagnosis is eligible for a study. Thus, our study may provide a less biased estimate of disparities for participation in PICU research by conditioning on patients actually eligible for the research. By including 42 total studies, both observational and interventional, our study evaluates the effects of multiple sociodemographic exposures across a variety of clinical and research scenarios. In general, even demographic groups with lower odds of consent were more likely to consent to interventional studies. Prior investigations on disparities in PICU research were limited to single-parent studies.^22,23^ Our study provides some additional nuance regarding the motivations of families affecting their willingness to have their critically ill child participate in research.

Our results highlight the effect of communication between research staff and families on equitable inclusion in research. Differential rates of being approached for research may reflect implicit bias of the research team or the clinical attending physician in assessing likelihood of consent or uncertainty in building rapport with the family. Disparities according to language may reflect inadequacy of or discomfort using interpreter services, limited parental presence at bedside, or accessibility by telephone. Effective interpreter use could mitigate the lower rates of approach seen for non-English speakers, especially Spanish speakers, who were approached at lower rates but had similar odds of consent relative to English speakers. By contrast, speakers of languages other than Arabic, English, or Spanish had the lowest odds of consenting even when approached, which may be due to inadequacy of communication even if approached. Institutional review board inclusion and exclusion criteria varied in their consideration of families with non–English language preference. Parental absence at bedside accounted for nearly half of cases of inability to approach families in the RESTORE trial^22^ and was also a factor in our cohort. Improved research staff training (including bias training), increased awareness of and access to interpretation services, availability of telephone consent, enrollment outside business hours (especially for studies not above minimal risk), greater use of video-conferencing technologies and web-based signatures, and deferring immediate consent in appropriate scenarios are all strategies to increase rates of approach.

Lower rates of approach for research participation may not, however, account for all of the observed disparities in study enrollment. Among Black children, half of the observed disparity in consent was mediated by the lower probability of being approached. The reasons why families of Black children are less likely to consent after being approached requires dedicated exploration. Solving this problem is essential for ensuring that conclusions from research are generalizable and applicable to the population at risk for the conditions being studied.

While our study offers some perspectives on disparities in PICU-based research, additional work is necessary. A survey of primary care clinicians found greater mistrust of research among Black parents.^29^ Attitudes toward research may differ in the intensive care setting, and existing studies can be affected by participation bias, limiting the utility of such studies to the PICU population. Survey studies carry the additional concerns of social desirability bias and acquiescence bias. Barriers to parental consent to PICU research after being approached may also include time constraints, feeling overwhelmed, perception of research being burdensome, health literacy, trust in the medical system, and research not being explained well.^23,30,31^ Variations in these attitudes by social or cultural group are not well understood and likely differ between institutions. Racism in the health care field, for example, varies geographically in the US.^16,32^ Additional research is needed to better understand why families are approached at varying frequencies and how a family decides whether to consent following approach, both globally and within specific institutions. Qualitative research methods may provide unique opportunities and insights to address these questions.

### Limitations

Our study has limitations. We were limited to review of consent logs from a single institution, and some findings may not generalize to all North American PICUs. However, our demographics and severity of illness are similar to many other academic PICUs engaged in research. Our study relied on documentation of race and ethnicity, language, and religion in the EMR, which may lead to exposure misclassifications if categories were entered inaccurately. Minority races and ethnicities are more likely to have discordance between EMR- and self-reported race and ethnicity,^33,34^ although a recent study assessing this in pediatrics found reasonably high overall concordance (κ = 0.77).^33^ Local patient demographics led to many racial and ethnic, linguistic, or religious groups being combined into groups designated other. This resulted in substantial heterogeneity within these groups and limited generalizability outside the larger sociodemographic groups we report herein. Overall, these limitations related to documentation of our exposure variables require our results to be interpreted cautiously and highlight the importance of future studies investigating disparities to standardize the recording of sociodemographic variables. The method in which we handled non-English language for eligibility (ie, eligible unless the study protocol excluded) was an effort to capture potential causes of disparities in approach and consent related to language, but this may introduce potential bias. Certain factors that may affect research participation, such as demographic characteristics of research coordinators, consenting parties, and medical teams, were not available. Last, while the availability of detailed screening logs helped to inform conclusions, we have only limited information about why patients were not approached and no information about why families declined.

## Conclusions

In this cohort study of consent rates for PICU research participation, we found lower odds of enrollment according to race and ethnicity, language, religion, and degree of social deprivation. These disparities were largely attributable to disparate rates of approach for research participation, with the important exception of Black children, who were less likely to be enrolled even after accounting for lower rates of approach. Future research should seek to better understand cultural attitudes toward pediatric research in the PICU and test interventions to improve communication and trust between research teams and families.

## Notes

Supplement 1. **eMethods.** Data Source, Eligibility, Definitions, Statistical Analysis **eFigure 1**. Directed Acyclic Graph Informing Regression Models **eFigure 2.** Flowchart of Study Participation **eTable 1.** Odds of Approach for Study Participation According to Race and Ethnicity, Preferred Language, Religion, or Social Deprivation Index **eTable 2.** Reasons for Not Approaching by Race and Ethnicity **eTable 3.** Reasons for Not Approaching by Language Preferred **eTable 4.** Reasons for Not Approaching by Religion **eTable 5.** Odds of Approach Stratified According to Study Type **eTable 6.** Odds of Consent for Study Participation Among All Eligible Patients According to Race and Ethnicity, Preferred Language, Religion, or Social Deprivation Index **eTable 7.** Odds of Consent Stratified According to Study Type **eTable 8.** Odds of Consent for Study Participation Among Approached Patients According to Race and Ethnicity, Preferred Language, Religion, or Social Deprivation Index **eTable 9.** Odds of Consent Restricted to Those Approached for a Study, Stratified According to Study Type **eTable 10.** Odds of Approach, Consent, and Consent Restricted to Those Approached for a Study, With Multiple Imputation of Missing Data **eTable 11.** Odds of Approach, Consent, and Consent Restricted to Those Approached for a Study, With All Exposures Included in the Same Model, Using the Dataset With Imputed Missing Variables **eTable 12.** Results of a Multinomial Logistic Regression for Odds of Approached and Declined Consent and Approached and Provided Consent, With Not Approached Used as the Reference

Supplement 2.
Data Sharing Statement
