# Supplementary material for: Racial and Ethnic Disparity in Approach for Pediatric Intensive Care Unit Research Participation
Source: JAMA Netw Open. 2024 May 15;7(5):e2411375. doi: 10.1001/jamanetworkopen.2024.11375 (PMC11096993; doi:10.1001/jamanetworkopen.2024.11375)
Supplement: Supplement 1. — eMethods. Data Source, Eligibility, Definitions, Statistical Analysis eFigure 1. Directed Acyclic Graph Informing Regression Models eFigure 2. Flowchart of Study Participation eTable 1. Odds of Approach for Study Participation According to Race and Ethnicity, Preferred Language, Religion, or Social Deprivation Index eTable 2. Reasons for Not Approaching by Race and Ethnicity eTable 3. Reasons for Not Approaching by Language Preferred eTable 4. Reasons for Not Approaching by Religion eTable 5. Odds of Approach Stratified According to Study Type eTable 6. Odds of Consent for Study Participation Among All Eligible Patients According to Race and Ethnicity, Preferred Language, Religion, or Social Deprivation Index eTable 7. Odds of Consent Stratified According to Study Type eTable 8. Odds of Consent for Study Participation Among Approached Patients According to Race and Ethnicity, Preferred Language, Religion, or Social Deprivation Index eTable 9. Odds of Consent Restricted to Those Approached for a Study, Stratified According to Study Type eTable 10. Odds of Approach, Consent, and Consent Restricted to Those Approached for a Study, With Multiple Imputation of Missing Data eTable 11. Odds of Approach, Consent, and Consent Restricted to Those Approached for a Study, With All Exposures Included in the Same Model, Using the Dataset With Imputed Missing Variables eTable 12. Results of a Multinomial Logistic Regression for Odds of Approached and Declined Consent and Approached and Provided Consent, With Not Approached Used as the Reference [file jamanetwopen-e2411375-s001.pdf]

Supplementary Online Content

Mayer SL, Brajcich MR, Juste L, Hsu JY, Yehya N. Racial and ethnic disparity in approach for pediatric intensive care unit research participation. *JAMA Netw Open*. 2024;7(5):e2411375. doi:10.1001/jamanetworkopen.2024.11375

eMethods. Data Source, Eligibility, Definitions, Statistical Analysis

eFigure 1. Directed Acyclic Graph Informing Regression Models

eFigure 2. Flowchart of Study Participation

eTable 1. Odds of Approach for Study Participation According to Race and Ethnicity, Preferred Language, Religion, or Social Deprivation Index

eTable 2. Reasons for Not Approaching by Race and Ethnicity

eTable 3. Reasons for Not Approaching by Language Preferred

eTable 4. Reasons for Not Approaching by Religion

eTable 5. Odds of Approach Stratified According to Study Type

eTable 6. Odds of Consent for Study Participation Among All Eligible Patients According to Race and Ethnicity, Preferred Language, Religion, or Social Deprivation Index

eTable 7. Odds of Consent Stratified According to Study Type

eTable 8. Odds of Consent for Study Participation Among Approached Patients According to Race and Ethnicity, Preferred Language, Religion, or Social Deprivation Index

eTable 9. Odds of Consent Restricted to Those Approached for a Study, Stratified According to Study Type

eTable 10. Odds of Approach, Consent, and Consent Restricted to Those Approached for a Study, With Multiple Imputation of Missing Data

eTable 11. Odds of Approach, Consent, and Consent Restricted to Those Approached for a Study, With All Exposures Included in the Same Model, Using the Dataset With Imputed Missing Variables

eTable 12. Results of a Multinomial Logistic Regression for Odds of Approached and Declined Consent and Approached and Provided Consent, With Not Approached Used as the Reference

This supplementary material has been provided by the authors to give readers additional information about their work.

© 2024 Mayer SL et al. *JAMA Network Open*.

eMethods. Data Source, Eligibility, Definitions, Statistical Analysis

Data Source

This was a retrospective cohort study that reviewed all screening and consent logs for all research studies prospectively enrolling in the Children's Hospital of Philadelphia (CHOP) PICU from July 2011 to December 2021. Screening logs were maintained by a dedicated team of research coordinators and research assistants housed in the Division of Critical Care Medicine that manage all patient-facing PICU research. All research materials are kept on dedicated password-protected shared drives on CHOP servers, which is itself subdivided by faculty principal investigator (PI). Each faculty folder holds all studies that the specific PI is responsible for that are housed in the PICU. Studies occurring in non-PICU locations (emergency department, cardiac ICU) but managed by PIs housed in the PICU are included in these logs.

Individual study folders include all data elements, including the protocol that had been approved by the CHOP Institutional Review Board (IRB), a master list linking patient-specific identifiable health information (i.e., medical record number) to detailed screening logs. These logs are updated in real-time as subjects are screened for eligibility, approached, and consented. Reasons for subjects being ineligible were recorded almost universally, and were based on the exclusion criteria of specific studies. Reasons for not approaching a subject were more variably recorded, but were available for the majority of subjects. Both reasons for ineligibility and reasons for not approaching a subject were generally recorded as free-text fields, but had consistent terminology within a specific study. For example, studies investigating sepsis that required at least two organ failures could have a subject deemed ineligible for "less than 2 organ failures" noted in their screening log.

© 2024 Mayer SL et al. *JAMA Network Open*.

The assigned study coordinator was responsible for maintaining the screening logs in real-time, and for ensuring their accuracy. If a coordinator left CHOP, responsibility for maintaining records was passed onto another coordinator. Ultimate responsibility for ensuring no loss of access to data over time lay with the manager for the coordinator group. Paper records (e.g., case report and consent forms) were eventually housed off-campus for studies as they closed; electronic records were maintained indefinitely on the shared research drive.

Generally, study coordinators and PIs met regularly (typically at a frequency of every 1 to 4 weeks, depending on the study and PI) to review eligibility, approaches, and consent rates. Access to the password-protected research drive, and to specific studies, was contingent upon being listed as personnel on the IRB for the specific study. For the purpose of our study, after our study received its own IRB approval, the individual coordinators and the PI of this study went through each PI sub-folder and extracted studies requiring prospective consent.

Eligibility

All subjects eligible for research requiring consent were potentially eligible for this study. Studies using exception from informed consent (emergency trials) or waiver of informed consent (data extraction only) were not included in our analysis. Study protocols for each study were examined for specific inclusion and exclusion criteria in order to determine eligibility for this study. All studies approached subjects without explicit consideration of race/ethnicity or gender. For the purposes of our analysis, families with non-English language preference were considered ineligible if the protocol for that study specified language requirements as an eligibility criterion. For example, some studies explicitly listed "English and Spanish-speaking

© 2024 Mayer SL et al. *JAMA Network Open*.

only" in their eligibility criteria, with specific consent forms only for those two languages; when assessing subjects from this study, a non-English non-Spanish speaking family would be (by definition) ineligible for our study. However, if the study protocol had no language limitation, subjects were considered potentially eligible for that study, and reasons for non-approach or non-consent (including those related to language) were recorded. In all cases, screening log entries for reasons for exclusion and reasons for not approaching were matched against the study protocol to ensure coherence. Entries with missing or ambiguous data that precluded a clear assessment of subject eligibility were excluded. For studies with time-sensitive enrollment, children noted to be "out of window" at screening were also excluded as ineligible. However, if specific staff or facility limitations outside of explicit research protocols (e.g., treating attending preference, research staff or PI discretion) prevented timely enrollment, children were included as eligible.

Definitions

Screening logs were linked to the electronic medical record (EMR) for data collection. The EMR contains detailed demographic data collected from dedicated administrators that record the information directly from families upon admission to the PICU using standardized intake questionnaires. We examined four distinct exposures: race/ethnicity, preferred language, religion, and Social Deprivation Index (SDI). Since our EMR permits Hispanic to be reported as either a race or ethnicity, we combined race and ethnicity into groupings of non-Hispanic White, non-Hispanic Black, Hispanic (any Hispanic code), and Other Race. Other Race consisted of Asian, Pacific Islander, Indian, Multiracial, Mixed, Other, and Refused.

© 2024 Mayer SL et al. *JAMA Network Open*.

Preferred language was encoded as English, Spanish, Arabic, and Other Language. Other Language included Bengali, Burmese, Cantonese, Creole, French, Fulani, Hindi, Nepali, Pashto, Portuguese, Punjabi, Russian, Swahili, Tagalog, Turkish, Urdu, and Vietnamese.

Religion was coded as None, Christian, Jewish, Muslim, and Other Religion. Christian grouped together Apostolic, Baptist, Catholic, Christian, Christian Non-Denominational, Episcopal, Evangelical, Jehovah's Witness, Lutheran, Mennonite, Methodist, Mormon, Pentecostal, Presbyterian, Protestant, Quaker, Roman Catholic, Seventh Day Adventist, and Unitarian. Other Religion included Baha'i, Buddhist, Hindu, Jainism, Other, Sikhism, and Wiccan.

Zip code at the time of eligibility was used to assign SDI, a validated composite metric of area-level deprivation based on seven demographic characteristics collected in the American Community Survey. SDI ranges from 0 (least) to 100 (most deprived), and was divided by ten for reporting in multivariable regression models.

We modeled three distinct outcomes: approach for research participation, consent to research study among all eligible subjects, and consent to research among those approached. Confounders included age, recorded sex, PICU length of stay (in days) prior to screening, illness severity as defined by Pediatric Risk of Mortality (PRISM) III score at 12 hours, year of screening, elective or non-elective admission, origin of admission (emergency department, inpatient floor, neonatal ICU, operating room, or outside hospital) and study type (observational or interventional). Entries reflecting the same patient eligible for different trials or on separate admissions were retained as separate encounters. Quantitative variables were treated as continuous variables.

© 2024 Mayer SL et al. *JAMA Network Open*.

Statistical Analysis

Separate multivariable logistic regression models were used to separately test the association between the four exposures of interest (race/ethnicity, language, religion, SDI) with three outcomes: approach for study (all eligible subjects), consent to study (all eligible subjects), and consent if approached for participation (restricted to those approached). All analyses were adjusted for confounders selected using a causal framework, and all analyses used robust variance estimators to account for two-way non-nested clustering (by subject and by study). Exposures had < 5% missingness except for language (6%), and as the non-random missingness of language made imputation conceptually difficult with the available variables, only complete case analyses were conducted in the primary analyses.

Exposures were analyzed in independent models given the complex interactions and potential collinearity between race/ethnicity, language, religion, and SDI (Supplementary Figure 1). Importantly, references to race and ethnicity in this manuscript are not meant to imply associations due to “biologic” (e.g., genetic) differences; rather, these exposures are meant to capture the lived experience of a given race or ethnicity, including of racism or bias.

We performed multiple additional analyses. First, given possible differences between observational and interventional studies, we *a priori* tested for differential associations between exposures and outcomes according to study type. Second, to test the impact of data missingness on our conclusions, we repeated analyses using multiple imputation by chained equations (10 imputations over the entire cohort) to impute missing values for language, religion, and SDI. Third, we performed an exploratory analysis by including all exposure variables in the model, in addition to confounders, on the dataset with imputed missing data.

© 2024 Mayer SL et al. *JAMA Network Open*.

Fourth, as an alternative method to model the data, we performed multinomial regression for odds of “approach and declining consent” and “approach and providing consent,” with “not approached” used as the reference.

Finally, causal mediation analysis was performed to estimate the degree to which the association between Black race and odds of consent was mediated by the probability of being approached (natural indirect effect). This was a two-step procedure where we initially modeled the predicted probability of being approached for all subjects using a regression model with all variables included as independent variables and using approach as the outcome. We then used this predicted probability of approach (a continuous variable that had values between 0 and 1) as a mediator of the association between Black race (versus White) and odds of consent. All analyses were conducted in Stata 18. We considered $p < 0.05$ as significant for main analyses, and $p < 0.10$ for assessing the significance of interaction terms.

© 2024 Mayer SL et al. *JAMA Network Open*.

eFigure 1. Directed Acyclic Graph Informing Regression Models

Note the inter-relatedness of the exposure variables (i.e., cyclic rather than acyclic), motivating the separate regression models for each exposure as the primary analyses.

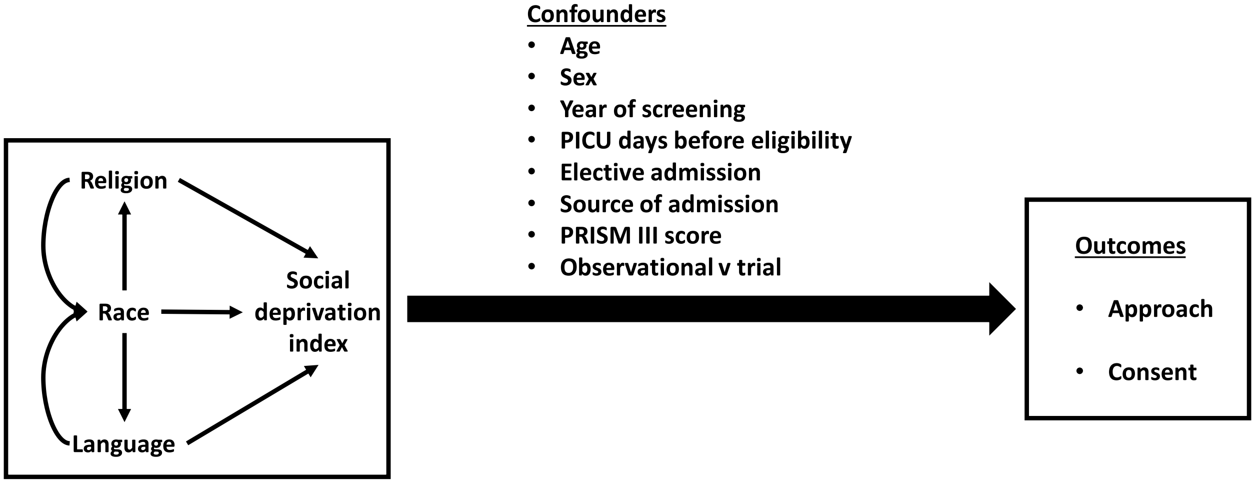

© 2024 Mayer SL et al. *JAMA Network Open*.

eFigure 2. Flowchart of Study Participation

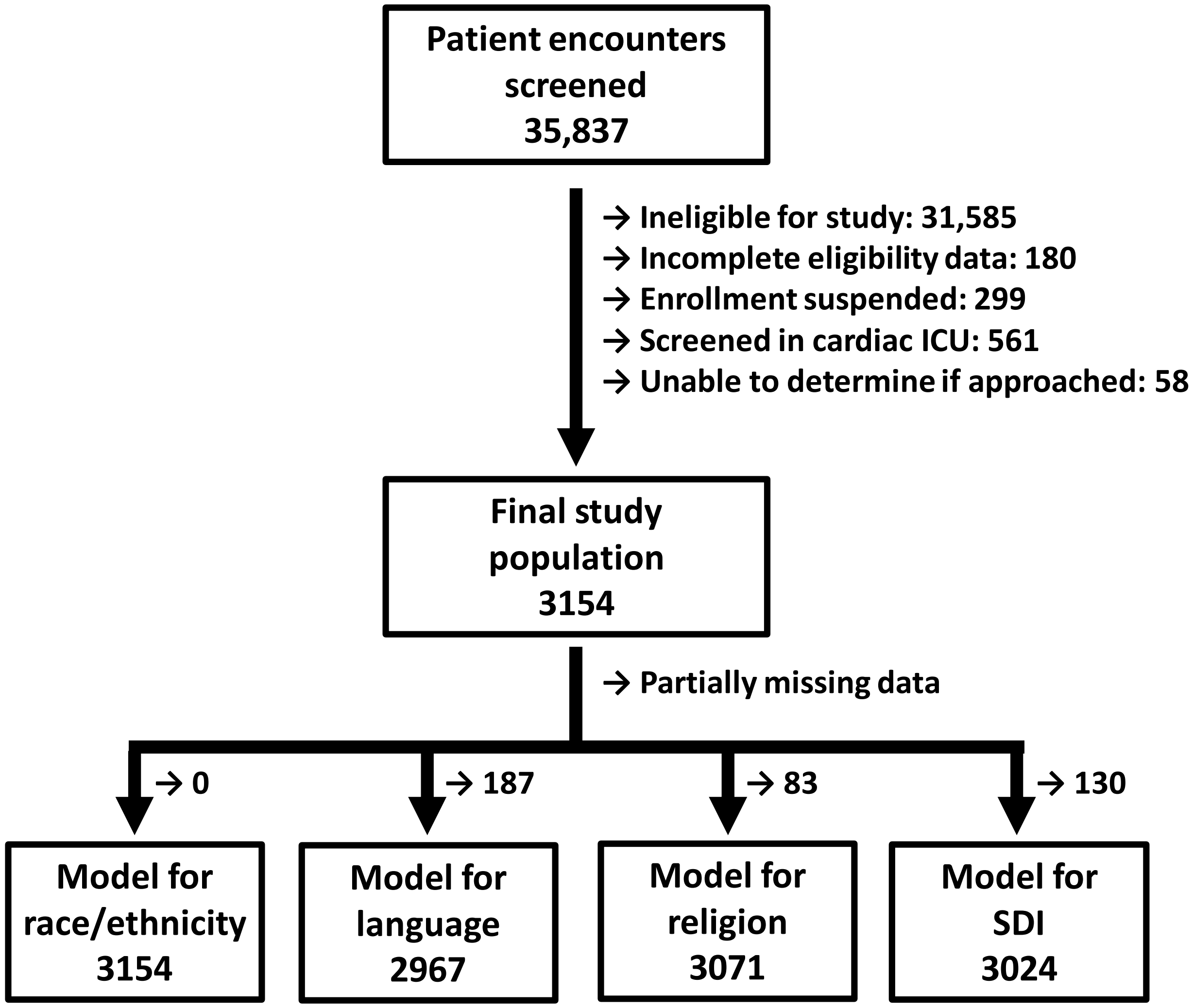

© 2024 Mayer SL et al. *JAMA Network Open*.

eTable 1. Odds of Approach for Study Participation According to Race and Ethnicity, Preferred Language, Religion, or Social Deprivation Index

Variable	Unadjusted	p value	Adjusted	p value
Race/ethnicity				
White	Ref	-	Ref	-
Black	**0.64 (0.52 to 0.79)**	**< 0.001**	**0.60 (0.49 to 0.73)**	**< 0.001**
Hispanic	**0.59 (0.44 to 0.80)**	**0.001**	**0.57 (0.42 to 0.76)**	**< 0.001**
Other	**0.47 (0.36 to 0.61)**	**< 0.001**	**0.44 (0.35 to 0.56)**	**< 0.001**
Language				
English	Ref	-	Ref	-
Spanish	**0.50 (0.29 to 0.85)**	**0.01**	0.57 (0.32 to 1.02)	0.06
Arabic	**0.28 (0.16 to 0.50)**	**< 0.001**	**0.28 (0.15 to 0.51)**	**< 0.001**
Other	**0.12 (0.07 to 0.22)**	**< 0.001**	**0.12 (0.07 to 0.21)**	**< 0.001**
Religion				
None	Ref	-	Ref	-
Christian	1.05 (0.83 to 1.34)	0.68	1.00 (0.81 to 1.23)	0.97
Muslim	**0.46 (0.32 to 0.66)**	**< 0.001**	**0.41 (0.28 to 0.59)**	**< 0.001**
Jewish	1.47 (0.94 to 2.29)	0.09	1.51 (0.94 to 2.43)	0.09
Other	0.91 (0.62 to 1.34)	0.62	0.79 (0.54 to 1.16)	0.23
Social deprivation index	**0.95 (0.92 to 0.97)**	**< 0.001**	**0.95 (0.93 to 0.98)**	**< 0.001**

Adjusted analyses are adjusted for age, sex, PICU length of stay before screening date, PRISM III score, year of screening, elective or non-elective admission, and origin of admission.

© 2024 Mayer SL et al. *JAMA Network Open*.

eTable 2. Reasons for Not Approaching by Race and Ethnicity

Reason	Non-Hispanic White (n = 254)	Non-Hispanic Black (n = 258)	Hispanic (n = 153)	Other (n = 231)
Clinical attending decline	59 (23)	42 (16)	19 (12)	32 (14)
Family unavailable	51 (20)	82 (32)	48 (31)	53 (23)
Perceived language barrier	5 (2)	4 (2)	34 (22)	75 (32)
Clinical staff unavailable	59 (23)	38 (15)	27 (18)	30 (13)
Other*	80 (31)	92 (36)	25 (16)	41 (18)

χ^2 174, *P* < .001

* Other includes previously declined all research, exceeded allowable phlebotomy limits, unclear legal situation or guardianship, study PI discretion, poor prognosis, and competing study.

© 2024 Mayer SL et al. *JAMA Network Open*.

eTable 3. Reasons for Not Approaching by Language Preferred

Reason	English (n = 666)	Spanish (n = 56)	Arabic (n = 59)	Other (n = 54)
Clinical attending decline	129 (19)	3 (5)	10 (17)	2 (4)
Family unavailable	189 (28)	17 (30)	9 (15)	4 (7)
Perceived language barrier	14 (2)	28 (50)	31 (53)	37 (69)
Clinical staff unavailable	127 (19)	3 (5)	8 (14)	5 (9)
Other*	207 (31)	5 (9)	1 (2)	6 (11)

χ^2 361, $P < 0.001$

* Other includes previously declined all research, exceeded allowable phlebotomy limits, unclear legal situation or guardianship, study PI discretion, poor prognosis, and competing study.

© 2024 Mayer SL et al. *JAMA Network Open*.

eTable 4. Reasons for Not Approaching by Religion

Reason	None (n = 187)	Christianity (n = 429)	Islam (n = 132)	Judaism (n = 21)	Other (n = 92)
Clinical attending decline	19 (10)	88 (21)	17 (13)	7 (33)	15 (16)
Family unavailable	61 (33)	101 (24)	28 (21)	3 (14)	32 (35)
Perceived language barrier	18 (10)	34 (8)	48 (36)	0	13 (14)
Clinical staff unavailable	44 (24)	74 (17)	12 (9)	1 (5)	17 (18)
Other*	45 (24)	132 (31)	27 (20)	10 (48)	15 (16)

χ^2 111, $P < 0.001$

* Other includes previously declined all research, exceeded allowable phlebotomy limits, unclear legal situation or guardianship, study PI discretion, poor prognosis, and competing study.

© 2024 Mayer SL et al. *JAMA Network Open*.

eTable 5. Odds of Approach Stratified According to Study Type

Variable	Observational	Interventional	Interaction p
Race/ethnicity			
White	Ref	Ref	-
Black	0.56 (0.44 to 0.71)	0.69 (0.53 to 0.89)	0.32
Hispanic	0.57 (0.39 to 0.86)	0.51 (0.41 to 0.64)	0.62
Other	0.41 (0.31 to 0.55)	0.54 (0.40 to 0.72)	0.19
Language			
English	Ref	Ref	-
Spanish	0.52 (0.26 to 1.07)	0.74 (0.36 to 1.54)	0.55
Arabic	0.23 (0.12 to 0.45)	0.46 (0.19 to 1.11)	0.29
Other	**0.09 (0.05 to 0.16)**	**0.25 (0.08 to 0.84)**	**0.09**
Religion			
None	Ref	Ref	-
Christian	0.95 (0.73 to 1.23)	1.12 (0.78 to 1.59)	0.63
Muslim	0.39 (0.25 to 0.59)	0.47 (0.24 to 0.91)	0.94
Jewish	1.28 (0.70 to 2.34)	2.55 (1.26 to 5.18)	0.25
Other	0.80 (0.50 to 1.29)	0.79 (0.49 to 1.27)	0.97
Social deprivation index	0.96 (0.93 to 0.99)	0.94 (0.91 to 0.98)	0.22

All analyses are adjusted for age, sex, PICU length of stay before screening date, PRISM III score, year of screening, elective or non-elective admission, and origin of admission.

© 2024 Mayer SL et al. *JAMA Network Open*.

eTable 6. Odds of Consent for Study Participation Among All Eligible Patients According to Race and Ethnicity, Preferred Language, Religion, or Social Deprivation Index

Variable	Unadjusted	p value	Adjusted	p value
Race/ethnicity				
White	Ref	-	Ref	-
Black	**0.65 (0.51 to 0.82)**	**< 0.001**	**0.59 (0.46 to 0.77)**	**< 0.001**
Hispanic	0.77 (0.56 to 1.07)	0.13	0.80 (0.59 to 1.10)	0.17
Other	**0.66 (0.50 to 0.86)**	**0.003**	**0.58 (0.42 to 0.79)**	**0.001**
Language				
English	Ref	-	Ref	-
Spanish	0.81 (0.52 to 1.26)	0.34	0.91 (0.57 to 1.45)	0.68
Arabic	**0.48 (0.27 to 0.87)**	**0.02**	**0.45 (0.24 to 0.85)**	**0.02**
Other	**0.15 (0.07 to 0.30)**	**< 0.001**	**0.14 (0.06 to 0.31)**	**< 0.001**
Religion				
None	Ref	-	Ref	-
Christian	0.97 (0.77 to 1.23)	0.81	1.04 (0.82 to 1.31)	0.77
Muslim	**0.56 (0.38 to 0.82)**	**0.003**	**0.56 (0.36 to 0.86)**	**0.009**
Jewish	0.75 (0.48 to 1.19)	0.23	0.75 (0.46 to 1.24)	0.26
Other	0.83 (0.59 to 1.18)	0.31	0.88 (0.62 to 1.24)	0.45
Social deprivation index	**0.97 (0.94 to 1.00)**	**0.03**	0.97 (0.94 to 1.01)	0.07

Adjusted analyses are adjusted for age, sex, PICU length of stay before screening date, PRISM III score, year of screening, elective or non-elective admission, and origin of admission.

© 2024 Mayer SL et al. *JAMA Network Open*.

eTable 7. Odds of Consent Stratified According to Study Type

Variable	Observational	Interventional	Interaction p
Race/ethnicity			
White	Ref	Ref	-
Black	0.57 (0.43 to 0.74)	0.67 (0.40 to 1.13)	0.42
Hispanic	0.77 (0.53 to 1.11)	0.84 (0.57 to 1.22)	0.46
Other	**0.51 (0.35 to 0.74)**	**0.83 (0.62 to 1.12)**	**0.009**
Language			
English	Ref	Ref	-
Spanish	0.85 (0.49 to 1.49)	1.11 (0.61 to 2.03)	0.57
Arabic	0.41 (0.18 to 0.92)	0.59 (0.24 to 1.45)	0.59
Other	**0.09 (0.04 to 0.21)**	**0.44 (0.14 to 1.41)**	**0.03**
Religion			
None	Ref	Ref	-
Christian	0.97 (0.74 to 1.25)	1.32 (0.84 to 2.08)	0.71
Muslim	0.51 (0.31 to 0.83)	0.80 (0.44 to 1.44)	0.48
Jewish	0.70 (0.40 to 1.21)	1.05 (0.44 to 2.47)	0.35
Other	0.85 (0.57 to 1.25)	1.03 (0.58 to 1.84)	0.95
Social deprivation index	0.97 (0.94 to 1.01)	0.97 (0.92 to 1.02)	0.75

All analyses are adjusted for age, sex, PICU length of stay before screening date, PRISM III score, year of screening, elective or non-elective admission, and origin of admission.

© 2024 Mayer SL et al. *JAMA Network Open*.

eTable 8. Odds of Consent for Study Participation Among Approached Patients According to Race and Ethnicity, Preferred Language, Religion, or Social Deprivation Index

Variable	Unadjusted	p value	Adjusted	p value
Race/ethnicity				
White	Ref	-	Ref	-
Black	**0.73 (0.55 to 0.97)**	**0.03**	**0.68 (0.49 to 0.93)**	**0.02**
Hispanic	1.01 (0.69 to 1.47)	0.97	1.13 (0.80 to 1.60)	0.50
Other	0.96 (0.69 to 1.35)	0.83	0.86 (0.57 to 1.29)	0.47
Language				
English	Ref	-	Ref	-
Spanish	1.38 (0.92 to 2.06)	0.12	1.46 (0.92 to 2.31)	0.11
Arabic	1.15 (0.52 to 2.54)	0.74	1.14 (0.51 to 2.52)	0.75
Other	0.46 (0.20 to 1.04)	0.06	0.41 (0.14 to 1.21)	0.11
Religion				
None	Ref	-	Ref	-
Christian	0.92 (0.70 to 1.21)	0.57	1.06 (0.80 to 1.40)	0.68
Muslim	0.83 (0.50 to 1.37)	0.46	0.94 (0.63 to 1.68)	0.83
Jewish	**0.56 (0.32 to 0.96)**	**0.03**	0.57 (0.31 to 1.04)	0.07
Other	0.83 (0.52 to 1.31)	0.42	1.00 (0.64 to 1.57)	0.99
Social deprivation index	0.99 (0.95 to 1.03)	0.58	0.99 (0.95 to 1.04)	0.82

Adjusted analyses are adjusted for age, sex, PICU length of stay before screening date, PRISM III score, year of screening, elective or non-elective admission, and origin of admission.

© 2024 Mayer SL et al. *JAMA Network Open*.

eTable 9. Odds of Consent Restricted to Those Approached for a Study, Stratified According to Study Type

Variable	Observational	Interventional	Interaction p
Race/ethnicity			
White	Ref	Ref	-
Black	0.66 (0.48 to 0.92)	0.72 (0.37 to 1.40)	0.49
Hispanic	1.02 (0.70 to 1.50)	1.37 (0.76 to 2.45)	0.25
Other	**0.76 (0.48 to 1.20)**	**1.19 (0.72 to 1.96)**	**0.07**
Language			
English	Ref	Ref	-
Spanish	1.39 (0.85 to 2.29)	1.90 (0.55 to 6.58)	0.91
Arabic	1.30 (0.39 to 4.34)	1.03 (0.32 to 3.28)	0.75
Other	**0.24 (0.07 to 0.87)**	**1.24 (0.47 to 3.26)**	**0.08**
Religion			
None	Ref	Ref	-
Christian	0.99 (0.72 to 1.36)	1.33 (0.76 to 2.32)	0.87
Muslim	0.84 (0.44 to 1.60)	1.49 (0.77 to 2.85)	0.22
Jewish	0.55 (0.28 to 1.08)	0.70 (0.25 to 1.94)	0.63
Other	0.95 (0.58 to 1.58)	1.20 (0.50 to 2.86)	0.98
Social deprivation index	0.99 (0.94 to 1.04)	1.00 (0.91 to 1.10)	0.47

All analyses are adjusted for age, sex, PICU length of stay before screening date, PRISM III score, year of screening, elective or non-elective admission, and origin of admission.

© 2024 Mayer SL et al. *JAMA Network Open*.

eTable 10. Odds of Approach, Consent, and Consent Restricted to Those Approached for a Study, With Multiple Imputation of Missing Data

Variable	OR for approach (all eligible)	OR for consent (all eligible)	OR for consent (if approached)
Race/ethnicity			
White	Ref	Ref	Ref
Black	**0.60 (0.48 to 0.74)**	**0.59 (0.49 to 0.71)**	**0.68 (0.55 to 0.84)**
Hispanic	**0.57 (0.44 to 0.73)**	**0.80 (0.65 to 1.00)**	1.13 (0.85 to 1.49)
Other	**0.44 (0.36 to 0.55)**	**0.58 (0.47 to 0.71)**	0.86 (0.67 to 1.11)
Language			
English	Ref	Ref	Ref
Spanish	**0.58 (0.39 to 0.85)**	0.91 (0.64 to 1.28)	1.44 (0.90 to 2.30)
Arabic	**0.31 (0.21 to 0.46)**	**0.53 (0.35 to 0.79)**	1.26 (0.71 to 2.23)
Other	**0.13 (0.08 to 0.22)**	**0.15 (0.07 to 0.31)**	0.44 (0.17 to 1.13)
Religion			
None	Ref	Ref	Ref
Christian	1.01 (0.83 to 1.25)	1.05 (0.88 to 1.06)	1.06 (0.85 to 1.32)
Muslim	**0.41 (0.31 to 0.56)**	**0.55 (0.41 to 0.74)**	0.91 (0.62 to 1.35)
Jewish	1.47 (0.89 to 2.42)	0.76 (0.51 to 1.13)	**0.57 (0.37 to 0.89)**
Other	0.80 (0.59 to 1.09)	0.88 (0.67 to 1.17)	1.00 (0.71 to 1.41)
Social deprivation index	**0.96 (0.93 to 0.98)**	**0.97 (0.95 to 1.00)**	0.99 (0.97 to 1.02)

All analyses are adjusted for age, sex, PICU length of stay before screening date, PRISM III score, year of screening, elective or non-elective admission, and origin of admission.

© 2024 Mayer SL et al. *JAMA Network Open*.

eTable 11. Odds of Approach, Consent, and Consent Restricted to Those Approached for a Study, With All Exposures Included in the Same Model, Using the Dataset With Imputed Missing Variables

Variable	OR for approach (all eligible)	OR for consent (all eligible)	OR for consent (if approached)
Race/ethnicity			
White	Ref	Ref	Ref
Black	**0.67 (0.52 to 0.85)**	**0.57 (0.46 to 0.70)**	**0.58 (0.45 to 0.75)**
Hispanic	**0.67 (0.50 to 0.90)**	0.80 (0.62 to 1.04)	0.98 (0.71 to 1.35)
Other	**0.70 (0.54 to 0.90)**	**0.72 (0.57 to 0.91)**	0.80 (0.61 to 1.06)
Language			
English	Ref	Ref	Ref
Spanish	0.66 (0.42 to 1.04)	0.82 (0.55 to 1.22)	1.10 (0.64 to 1.90)
Arabic	**0.52 (0.31 to 0.89)**	0.74 (0.43 to 1.28)	1.37 (0.69 to 2.74)
Other	**0.16 (0.09 to 0.27)**	**0.18 (0.08 to 0.38)**	0.48 (0.17 to 1.32)
Religion			
None	Ref	Ref	Ref
Christian	1.00 (0.81 to 1.23)	1.02 (0.85 to 1.23)	1.05 (0.84 to 1.30)
Muslim	**0.60 (0.42 to 0.86)**	0.74 (0.51 to 1.07)	1.00 (0.63 to 1.59)
Jewish	1.26 (0.75 to 2.11)	**0.62 (0.41 to 0.94)**	**0.48 (0.30 to 0.77)**
Other	0.83 (0.60 to 1.15)	0.90 (0.68 to 1.21)	1.02 (0.72 to 1.44)
Social deprivation index	0.99 (0.96 to 1.02)	1.01 (0.98 to 1.04)	1.02 (0.99 to 1.06)

All analyses are adjusted for age, sex, PICU length of stay before screening date, PRISM III score, year of screening, elective or non-elective admission, and origin of admission. We caution that this approach does not have a causal interpretation.

© 2024 Mayer SL et al. *JAMA Network Open*.

eTable 12. Results of a Multinomial Logistic Regression for Odds of Approached and Declined Consent and Approached and Provided Consent, With Not Approached Used as the Reference

Variable	RR for approached and declined consent (versus not approached)	RR for approached and gave consent (versus not approached)	Between outcome group p
Race/ethnicity			
White	Ref	Ref	-
Black	**0.76 (0.59 to 0.97)**	**0.51 (0.41 to 0.65)**	**< 0.001**
Hispanic	**0.52 (0.38 to 0.72)**	**0.59 (0.45 to 0.77)**	0.39
Other	**0.50 (0.38 to 0.65)**	**0.42 (0.33 to 0.53)**	0.18
Language			
English	Ref	Ref	-
Spanish	**0.44 (0.26 to 0.74)**	**0.64 (0.43 to 0.96)**	0.11
Arabic	**0.27 (0.16 to 0.47)**	**0.28 (0.18 to 0.44)**	0.91
Other	**0.20 (0.10 to 0.38)**	**0.08 (0.04 to 0.17)**	0.06
Religion			
None	Ref	Ref	-
Christian	0.96 (0.75 to 1.24)	1.02 (0.81 to 1.27)	0.64
Muslim	**0.44 (0.30 to 0.64)**	**0.39 (0.28 to 0.55)**	0.59
Jewish	**2.10 (1.20 to 3.64)**	1.18 (0.68 to 2.03)	**0.01**
Other	0.80 (0.55 to 1.17)	0.79 (0.56 to 1.10)	0.90
Social deprivation index	**0.96 (0.93 to 0.99)**	**0.95 (0.93 to 0.98)**	0.69

All analyses are adjusted for age, sex, PICU length of stay before screening date, PRISM III score, year of screening, elective or non-elective admission, and origin of admission. The p value reports the results testing whether the association between an independent variable (e.g., Black race) and "approach and declining consent" differs significantly from the association with "approach and providing consent."

© 2024 Mayer SL et al. *JAMA Network Open*.
