# Supplementary material for: Racial and Ethnic Disparity in Approach for Pediatric Intensive Care Unit Research Participation
Source: JAMA Netw Open. 2024 May 15;7(5):e2411375. doi: 10.1001/jamanetworkopen.2024.11375 (PMC11096993; doi:10.1001/jamanetworkopen.2024.11375)
Supplement: Supplement 2. — Data Sharing Statement [file jamanetwopen-e2411375-s002.pdf]

Data Sharing Statement

Mayer. Racial and Ethnic Disparity in Approach for Pediatric Intensive Care Unit Research Participation. *JAMA Netw Open*. Published May 15, 2024.
doi:10.1001/jamanetworkopen.2024.11375

Data
Data available: Yes
Data types: Deidentified participant data
How to access data: Deidentified data upon request pursuant to regulations.
When available: With publication

Supporting Documents
Document types: None

Additional Information
Who can access the data: Qualified researchers
Types of analyses: re-analyses
Mechanisms of data availability: signed DUA
